# Psychological outcomes and support in grandparents whose grandchildren suffer from a severe physical illness: A systematic review

**DOI:** 10.1016/j.heliyon.2022.e09365

**Published:** 2022-05-06

**Authors:** Cristina Priboi, Barbara Gantner, Pauline Holmer, Luisa Neves da Silva, Eva Maria Tinner, Katharina Roser, Gisela Michel

**Affiliations:** a University of Lucerne, Frohburgstrasse 3, 6002, Lucerne, Switzerland; b Lucerne Cantonal Hospital, Spitalstrasse 6000, 16 Lucerne, Switzerland; c Bern University Hospital, Freiburgstrasse 18, 3010, Bern, Switzerland; d Cantonal Hospital Baselland, Rheinstrasse 26, 4410, Liestal, Switzerland; e University of Basel, Petersplatz 1, 4001, Basel, Switzerland

**Keywords:** Grandparent, Severe illness, Psychological outcomes, Support

## Abstract

**Objective:**

When a child is facing a severe physical illness, the entire family is affected. Grandparents provide invaluable emotional and practical support to families dealing with this situation, but little is known about the psychological impact on them. We aimed to synthesize the evidence on 1) the psychological outcomes experienced by grandparents when a grandchild is seriously ill and 2) the psychological support needed and used by grandparents.

**Methods:**

We systematically searched four databases with the search terms “grandchild”, “grandparents”, “psychological outcomes” and “severe diseases”, and we used narrative synthesis to analyze the extracted data.

**Results:**

Our search identified 3319 records of which 12 were included in the analysis. Grandparents reported experiencing a wide spectrum of feelings, with fear being the most prevalent feeling. Grandparents rarely accessed professional services due to their lack of knowledge about available programs or because of the absence of formal services addressing their needs. In consequence, grandparents asked and received informal support from other family members, friends or their church community.

**Conclusion:**

Grandparents need to be better informed about their grandchild's disease and the available support services in order to reduce their psychological burden and to better attend to the needs of the other family members.

## Highlights

• Grandparents' psychological wellbeing was highly affected by the illness of their grandchild.
• Grandparents experienced a roller-coaster of emotions, but the most predominant feeling was fear.
• Grandparents reported concerns for the ill grandchild, the parents, the siblings and themselves.
• Grandparents struggled with the lack of information about the child's illness.
• Grandparents rarely used formal help and were rather supported by non-professionals.

## 1 Introduction

Grandparents play an important part in the lives of their grandchildren. They are a valuable source of emotional and instrumental support for parents and grandchildren in general, but especially when a child is suffering from a severe illness [1]. When a child is diagnosed with a severe health condition or faces a life-threatening illness, the entire family is affected. Parents may suffer from increased psychological distress and anxiety, alongside burnout symptoms and overwhelming emotional experiences [2, 3, 4, 5, 6]. Besides parents, having a severely ill brother or sister, can also affect the psychological development and the mental health of the siblings. They tend to internalize their emotions, experience separation anxiety, report somatic complains and have sleeping problems [7]. The critical situation impacts not only the psychologic functioning of the family members, but also parents' work life, the economic situation of the family and the family's daily routine [8, 9]. There is some evidence showing that in these situations, grandparents step in and help the family by taking care of the ill child or by supervising the healthy siblings at home [10]. They provide moral support for the overwhelmed parents, take care of siblings and/or the ill child and sometimes even offer financial support in order to cover for disease related expenses [11, 12]. Parents identified grandparents as the first people they seek help from during challenging times [13] and they were also the first ones informed about the illness of the child [14, 15].

### 1.1 Grandparents’ emotional burden

Although grandparents often try to be strong for the family and consider their duty to take care of all family members, they are emotionally affected by the disease of the child as well. Similar to parents’ experience, the idea of losing a child causes stress and anxiety to grandparents [16, 17]. Learning about the diagnosis of their grandchild puts grandparents through a multitude of negative emotions, such as shock, fear, aggressiveness and powerlessness [14, 18].

### 1.2 Grandparents’ need for support

The extensive emotional burden experienced by grandparents creates a high need for emotional support. However, they tend to not acknowledge their own needs and their right to access support, especially when comparing themselves with the parents of the ill child [17]. Instead, grandparents often rely on their own partners or the parents of the ill child for emotional help by sharing painful and happy moments together and mutually support each other throughout the illness of the child [12, 14].

### 1.3 Objectives

The objective of this paper was to synthesize the literature exploring the psychological impact on grandparents whose grandchildren are suffering from a severe illness. Specifically, our aims were to describe:

i. The psychological outcomes experienced by grandparents who have a grandchild with a severe physical illness
ii. The psychological support needed and used by these grandparents.

## 2 Methods

The review protocol was registered on PROSPERO (No. CRD42021253500).

### 2.1 Search strategy

We searched four electronic databases PubMed, PsycINFO, CINAHL and Scopus on 22 March 2021 and updated the search on 19 October 2021, using four blocks of search terms: child, grandparent, psychological outcomes and severe illness (Appendix). A list of the most common pediatric illnesses was created in collaboration with a multidisciplinary team of experts consisting of three psychologists and two medical doctors.

### 2.2 Eligibility criteria

We included articles with all types of study designs presented in peer-reviewed journals with focus on grandparents of children suffering from a severe physical illness. Eligible grandchildren were children facing a severe physical illness at the moment of data collection or who had suffered from a serious physical illness in the past and were cured at data collection. We included only children who experienced the severe illness before the age of 21. The disease of the grandchild had to be: i. physical, not mental; ii. potentially life-threatening and; iii. lead to prolonged and/or multiple hospitalizations. Health conditions with no existing curative treatment were not considered eligible, therefore studies on end-of-life patients were excluded from the present review. We also excluded studies where grandparents were the primary caregivers of the children, those which focused on other aspects than the psychological experiences of grandparents, which did not use self-reported data or which reported information on the family members in general, but not specifically on grandparents. We did not apply restrictions on sample sizes, language or time.

### 2.3 Study selection

All records found by the electronic search were compiled in an EndNote library and duplicates were removed. Two reviewers (CP, BG) independently assessed the eligibility of the articles by screening titles/abstracts followed by full-text screening of the remaining studies. If disagreement occurred, consensus was reached by discussion or a third reviewer (GM) was consulted. In case of uncertainty regarding the medical diagnosis, the two medical collaborators were included in the final decision.

### 2.4 Quality assessment

We used QATSDD for the quality assessment of the included articles [21]. The tool consists of maximum 16 items and is suitable for qualitative, quantitative and mixed-methods study designs. Two independent reviewers (CP, PH) assessed the evaluation criteria on a scale from 0 to 3 and a percentage of the maximum score was calculated for each paper.

### 2.5 Data extraction

Data extraction was conducted by CP and double-checked by PH. Agreement was reached by discussions. The following information regarding the study characteristics was selected from the included articles: study origin, study design, aims, used measurements and the time span of data collection. Concerning participants’ characteristics, we separately extracted information on the grandparents of the severely ill children, comparison group, if included, and the ill children themselves. In terms of grandparents and comparison population, the sample size, gender, age, occupation, education and marital status were extracted, while for the grandchildren population, we extracted the type of diagnosis, treatment, age at data collection, age at diagnosis and gender.

### 2.6 Analysis

Due to the inclusion of quantitative and qualitative studies in the current review, we decided to use narrative synthesis to analyze and report the findings [22]. The outcomes of interest presented in each article were selected and categorized according to the two aims, psychological outcomes and psychological support. After the preliminary synthesis, the results from each section were inductively classified in subtopics by conducting thematic analysis in order to get a better overview of the findings. In a subsequent step, we compared the key subtopics between studies and we identified existing patterns and relationships across studies.

## 3 Results

### 3.1 Study selection

**Figure 1:**
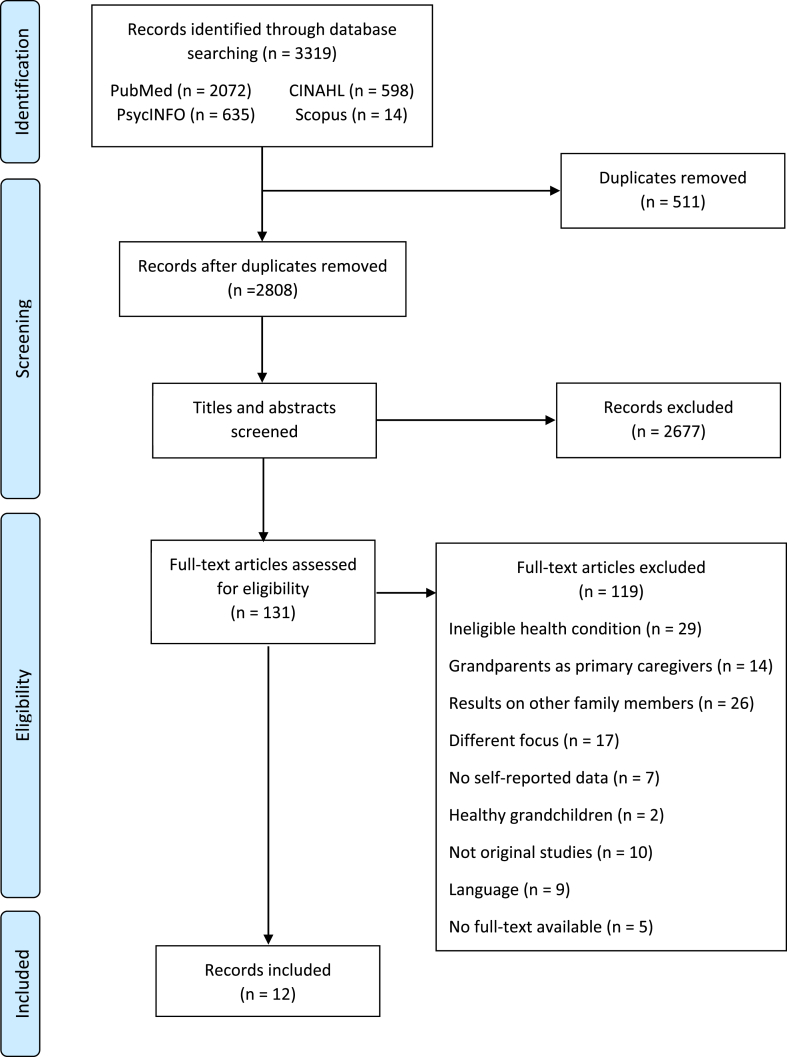
PRISMA flow diagram.

A total of 3319 records were identified in the four searched databases: PubMed (n = 2072), PsycINFO (n = 635), CINAHL (n = 598) and Scopus (n = 14). After removing duplicates (n = 511), 2808 records were included in the title and abstract screening, and thereof, 131 in the full-text screening. Finally, 12 articles were included in the current review (Figure 1). The overall quality of the included articles was adequate ranging from 68% to 89% (Table 1) and the inter-rater reliability showed substantial agreement between reviewers with weighted Kappa = 0.736.

**Table 1:** Description of included articles.

Article	Article characteristics		Key findings
Blackburn & Lowen (1986) [18]	**Origin:** • USA **Illness:** • Premature birth **Sample:** • Grandparents (n = 83) ⁃ Gender ○ Female = 57 ○ Male = 26 ⁃ Age grandmothers ○ Range = 47-78 ○ Mean = 58.4 ○ SD^a^ = N/A^b^ ⁃ Age grandfathers ○ Range = 53-73 ○ Mean = 61.7 ○ SD = N/A • Comparison group – parents of the premature infants (n = 50) ⁃ Gender ○ Female = 36 ○ Male = 14 ⁃ Age mothers ○ Range = 20-38 ○ Mean = 31.1 ○ SD = N/A ⁃ Age fathers ○ Range = 22-43 ○ Mean = 32.4 ○ SD = N/A	**Design:** • Quantitative design • Cross-sectional study **Measurements:** • Open-ended and fixed-response items • List of emotions on a 5-point Likert scale (1 = did not experience feeling & 5 = experienced very intensely) **Analysis:** • Content analysis for the open-ended questions • Descriptive statistics for fixed-response items **Quality assessment:** • 73%	**Psychological outcomes:** • Grandparents reported a wide spectrum of emotions: shock when seeing the infant for the first time; anger towards the hospital visiting activity; fear; anxiety; helplessness, frustration, grief etc. • Grandparents reported experiencing multiple concerns: ⁃ for the infant's parents (70%), ⁃ for the premature child (N/A) ⁃ for themselves (49% grandmothers & 23% grandfathers) • Grandmothers experienced emotions stronger than grandfathers **Psychological support:** • Grandparents reported that their spouses were the main source of emotional support • Both grandmothers and grandfathers identified the most helpful source of information as being the infant's mother followed by the infant's father
Charlebois & Bouchard (2007) [14]	**Origin:** • Canada **Illness:** • Childhood cancer **Sample:** • Grandparents (n = 8) ⁃ Gender ○ Female = 5 ○ Male = 3 ⁃ Age grandparents ○ Range = 56-69 ○ Mean = N/A ○ SD = N/A • Comparison group – none	**Design:** • Qualitative design • Cross-sectional study **Measurements:** • Semi-structure interviews **Analysis:** • Thematic analysis **Quality assessment:** • 71%	**Psychological outcomes:** • Grandparents reported a wide spectrum of emotions: sadness, powerless, fear, concern, overwhelm, anger, rage, sense of injustice etc. • Grandparents reported feeling concerned for themselves, the parents, the ill grandchild and the other grandchildren • Grandparents silenced their suffering in order to protect their own children, the parents of the cancer ill child • Grandmothers reported more signs of being affected than grandfathers **Psychological support:** • All grandparents emphasized the importance of hope and of feeling supported by others in order to carry on • Grandparents used different support strategies: ⁃ Confidence in someone ⁃ Believe in something: medicine or religion ⁃ Get informed about the situation: three grandparents used the internet to educate themselves ⁃ Rationalize the situation by searching for “solutions”, “tools”, “logical elements” ⁃ Living in the present: “day to day”, “step by step”
Dias & Mendes-Castillo (2021) [25]	**Origin:** • Brazil **Illness:** • Childhood cancer **Sample:** • Grandparents (n = 11) ⁃ Gender ○ Female = 9 ○ Male = 2 ⁃ Age grandparents ○ Range = N/A ○ Mean = N/A ○ SD = N/A • Comparison group – none	**Design:** • Qualitative design • Cross-sectional study **Measurements:** • Semi-structured interviews **Analysis:** • Hybrid Thematic Analysis Framework **Quality assessment:** • 74%	**Psychological outcomes:** • Grandparents reported being deeply worried about the diagnosis of their grandchild and described various emotions: fear, anxiety, suffering, dissatisfaction and tolerance • Grandparents were struggling understanding what was happening, but at the same time were afraid to ask questions and be ignored or not be able to understand • Grandparents described ambivalent feelings about the health professionals. Some reported that the language used by the health professionals was difficult to understand and increased their suffering, but also created a barrier for understanding and helping their grandchild • Grandparents expressed their high need to help the parents and the grandchild, therefore offered extensive support to the family, but were careful not to overstep • Grandparents reported that their physical and mental health were affected by the disease of the child **Psychological support:** • Grandparents shared their suffering with the parents of the ill child, but did not want to overload the parents and the other family members • Praying themselves, but also asking other people to pray for their grandchild, helped grandparents believe that the ill child will get better • Seeing the disease as an opportunity to learn, improve and further develop themselves as human beings offered grandparents some comfort • Grandparents reported that the “support house” organized in the hospital help them throughout the treatment of their grandchild, by fostering contact and exchange with the families of other cancer ill children
Frisman et al. (2012) [23]	**Origin:** • Sweden **Illness:** • Premature birth **Sample:** • Grandparents (n = 11) ⁃ Gender ○ Female = 11 ○ Male = 0 ⁃ Age grandmothers ○ Range = 52-66 ○ Mean = N/A ○ SD = N/A • Comparison group – none	**Design:** • Qualitative design • Cross-sectional study **Measurements:** • Structured interviews **Analysis:** • Content analysis **Quality assessment:** • 83%	**Psychological outcomes:** • The news about the baby being prematurely born made grandmothers feel in shock and completely unprepared • Grandmothers experienced ambivalent feelings – joy of becoming a grandmother, but also fear for the future of the infant • Grandmothers experiences multiple concerns. They were worried about the mother's health, the infant's health and the parents' well-being • Grandmothers were reluctant in asking the parents questions in order not to worry or burden them **Psychological support:** • None of the grandmothers asked for professional support, but some communicated and talking about their feelings with relatives, friends and/or work colleagues • Receiving information about the health status of the child and trusting the medical personal reduced grandmothers' worries
Hall (2004) [20]	**Origin:** • Denmark **Illness:** • Critically ill children who received intensive care • Mixed diagnoses **Sample:** • Grandparents (n = 7) ⁃ Gender ○ Female = 0 ○ Male = 7 ⁃ Age grandfathers ○ Range = 56-66 ○ Mean = N/A ○ SD = N/A • Comparison group – none	**Design:** • Qualitative design • Cross-sectional study **Measurements:** • Semi-structured interviews **Analysis:** • Hermeneutics **Quality assessment:** • 74%	**Psychological outcomes:** • Grandfathers reported a variety of emotions: fear, helpless, anxiety, impatience, disappointed, insecurity • Grandfathers felt ambivalent feelings – happiness of becoming a grandfather, but at the same time worried about the infant's wellbeing • Grandfathers expressed being doubled concerned: ⁃ for the ill infant and ⁃ for their own children – the parents of the infant • Grandfathers were so overwhelmed and worried that their daily life and work got affected. They also reported being much more worried than they showed to the others **Psychological support:** • Receiving information about infant's state helped grandfathers coping better with the stressful situation • Grandfathers were consoled by knowing that other grandparents were in a similar situation and by feeling cared for by others (the parents, neighbors, friends and colleagues)
Hall (2004) [19]	**Origin:** • Denmark **Illness:** • Critically ill children who received intensive care • Mixed diagnoses **Sample:** • Grandparents (n = 7) ⁃ Gender ○ Female = 7 ○ Male = 0 ⁃ Age grandmothers ○ Range = 51-61 ○ Mean = N/A ○ SD = N/A • Comparison group – none	**Design:** • Qualitative design • Cross-sectional study **Measurements:** • Semi-structured interviews **Analysis:** • Hermeneutics **Quality assessment:** • 80%	**Psychological outcomes:** • Grandmothers felt scared, worried, frustrated, afraid, confused, nervous, helpless, powerless • Grandmothers also reported ambivalent feelings – joy of becoming a grandmother, but were reserved in their joy • Grandmothers experiences multiple concerns. They worried about the parents' wellbeing and their health and at the same time were insecure about the survival of the infant and his/her future development • Grandmothers felt the urge to help their own children and to follow what was happening with the infant **Psychological support:** • The main psychological support for grandmothers were the parents of the infant. They shared painful and happy moments together and “took turns to care for each other”
Mendes-Castillo & Bousso (2016) [11]	**Origin:** • Brazil **Illness:** • Childhood cancer **Sample:** • Grandmothers (n = 8) ⁃ Gender ○ Female = 8 ○ Male = 0 ⁃ Age grandmothers ○ Range = N/A ○ Mean = N/A ○ SD = N/A • Comparison group – none	**Design:** • Qualitative design • Cross-sectional study **Measurements:** • Unstructured interviews **Analysis:** • Hermeneutics **Quality assessment:** • 68%	**Psychological outcomes:** • Grandmothers reported a wide range of feeling: shock when learning about the disease, overwhelm, fear, helplessness and pride in how the parents of the child were handling the situation. • Grandmothers described multiple concerns. They were suffering for the ill child, the parents of the child, the siblings, themselves and the family as a whole • Grandmothers did not feel entitled to suffer and decided to suppress their feelings in front of the parents (“what suffering can be greater than that of the parents?”) • The disease of their grandchild made them “cherish the good moments with more intensity” **Psychological support:** • Some grandmothers discussed about their experiences with external people for comfort, while others received comfort from their own children – the parents of the ill child
Moules et al. (2012) [24]	**Origin:** • Canada **Illness:** • Childhood cancer **Sample:** • Grandparents (n = 16) ⁃ Gender ○ Female = 12 ○ Male = 4 ⁃ Age grandparents ○ Range = N/A ○ Mean = N/A ○ SD = N/A • Comparison group – none	**Design:** • Qualitative design • Cross-sectional study **Measurements:** • Unstructured interviews **Analysis:** • Hermeneutics **Quality assessment:** • 77%	**Psychological outcomes:** • When learning about the grandchild's disease, grandparents experienced feelings of shock and disbelief • Grandparents reported a wide range of feelings: ⁃ Lost their sense of security in the world and questioned the unfairness of the situation – “why her/him?” ⁃ Developed a sense of generosity and kindness ⁃ Fear for the grandchild's life (“sword of Damocles”) ⁃ Sense of helplessness ⁃ Anxiety as a result of their expectation to receive bad news ⁃ Proud of their own children in how they were handling the situation • Some grandparents become closer to the ill child and the parents, but felt guilty of being less involved with their other children and grandchildren • Grandparents were afraid of being intrusive and felt like they need to be caution to boundaries • Grandparents decided to silence their worries in order to not upset their own children and did not ask any questions. They relied on second hand information provided by the parents and described this situation as making them felt like constantly waiting and not knowing **Psychological support:** • One grandmother stated that visiting a support group could have had a cathartic role throughout the grandchild's disease (“a place to go and break down… and then go back to their kids to be a rock”)
Moules et al. (2012) [12]	**Origin:** • Canada **Illness:** • Childhood cancer **Sample:** • Grandparents (n = 16) ⁃ Gender ○ Female = 12 ○ Male = 4 ⁃ Age grandparents ○ Range = N/A ○ Mean = N/A ○ SD = N/A • Comparison group – none	**Design:** • Qualitative design • Cross-sectional study **Measurements:** • Unstructured interviews **Analysis:** • Hermeneutics **Quality assessment:** • 77%	**Psychological outcomes:** • Grandparents felt helpless, described themselves as bystanders and reported being unsure if they did or say the right thing • Grandparents were afraid of burdening their own children, therefore they withholding their worries • Grandparents experienced double concerns. They suffered and were worried for the ill grandchild, but also for the parents • Grandparents described changes in the family boundaries ⁃ Some become emotionally closer to their own children – “strengthened what was already there” ⁃ Other grandparents encountered disruption with external family members ⁃ Some grandparents reported developing difficulties in their relationship with their partner **Psychological support:** • Grandparents received different type of support, from the church, friends, immediate family (sisters, brothers) or their workplace (flexibility of employers), but their main source of support were the spouses. The relationships between grandparents were challenged by the situation, but many found strength and support from their partner • Grandparents described that there was no infrastructure available for them (e.g. psychologist, support group etc.) • Grandparents struggled with the lack of information and many suggested brochures or online sites as would have being useful
Ravindran & Rempel (2010) [15]	**Origin:** • Canada **Illness:** • Hypoplastic left heart syndrome **Sample:** • Grandparents (n = 15) ⁃ Gender ○ Female = 10 ○ Male = 5 ⁃ Age grandparents ○ Range = 50-68 ○ Mean = N/A ○ SD = N/A • Comparison group – none	**Design:** • Qualitative design • Cross-sectional study **Measurements:** • Semi-structured interviews **Analysis:** • Line-by-line open coding followed by open code categories **Quality assessment:** • 69%	**Psychological outcomes:** • Grandparents described triple concerns. They were worried about the parents, the ill child and also about the siblings of the ill child • Grandparents felt very proud about the siblings' adjustment to the entire stressful situation
Wakefield (2014) [17]	**Origin:** • Australia **Illness:** • Childhood cancer **Sample:** • Grandparents (n = 87)^c^ ⁃ Gender ○ Female = 57 ○ Male = 27 ⁃ Age grandparents ○ Range = 46-81 ○ Mean = 65.02 ○ SD = 6.6 • Comparison group –grandparents of health children (n = 134) ⁃ Gender ○ Female = 87 ○ Male = 47 ⁃ Age grandparents ○ Range = 45-87 ○ Mean = 65.75 ○ SD = 7.21	**Design:** • Mixed-methods design Cross-sectional study **Measurements:** • Emotion thermometers tool for distress, anxiety, depression, anger and need for help • Support usage: grandparents reported all support they used since becoming a grandparent (formal, semi-formal and informal) • Barriers: grandparents assessed 10 potential barriers to access support on a 4 point Likert scale • 3 open ended questions about the impact of cancer on grandparents' physical/emotional health, family roles relationships **Analysis:** • Various statistical analyzes were used – t-tests, chi-square, regression models **Quality assessment:** • 88%	**Psychological outcomes:** • Grandparents described going through a wide range of feelings, which led them feeling exhausted • Grandparents talked about the improved relationships within the family as a result of their grandchild's disease • Grandparents described difficulties coping with the uncertainty of the child's prognosis and the parent's distress • When compared with grandparents of health children, grandparents of cancer sick children showed higher levels of distress, anxiety, depression, anger and need for help than • Being a grandmother and having fewer grandchildren were identified as predictors for higher distress **Psychological support:** • Very few grandparents accessed professional psychological support. They used the church or religious groups instead and identified lack of knowledge and geographical isolation as barriers to attend professional support
Wakefield (2016) [16]	**Origin:** • Australia **Illness:** • Childhood cancer **Sample:** • Grandparents (n = 89) ⁃ Gender ○ Female = 56 ○ Male = 33 ⁃ Age grandparents ○ Range = 44-83 ○ Mean = 65.9 ○ SD = 7.7 • Comparison group –grandparents of health children (n = 133) ⁃ Gender ○ Female = 93 ○ Male = 40 ⁃ Age grandparents ○ Range = 43-83 ○ Mean = 67.3 ○ SD = 6.5	**Design:** • Quantitative design • Cross-sectional study **Measurements:** • QOL^d^ ○ WHOQOL-BREF^e^ ○ EQ-3D-5L^f^ • Open questions about relationship changes with grandchild or grandchild's family • Sleep quality – adaptation of Pittsburgh Sleep Quality Index • Medications/hospitalizations: ○ Grandparents listed all medications they had taken in the last 4 weeks ○ 4 purposely designed items assessed hospitalization **Analysis:** • Various statistical analyzes were used – t-tests, chi-square, regression models **Quality assessment:** • 89%	**Psychological outcomes:** • Grandparents of children with cancer reported lower levels of psychological health and higher levels of anxiety and depression compare to grandparents of healthy children • Predictors associated with lower QOL: ⁃ Being a grandmother ⁃ Living in a major city or an urban location ⁃ Being unemployed/retired ⁃ Living further from the grandchild ⁃ Having a female grandchild • Some grandparents described changes in the family relationships associated with the disease of the child. They become closer to their families, but also encountered problems in the relationship with their spouses, due to the fact that they spent too much time with the ill grandchild and the family

a Standard deviation.

b Not available.

c The difference between the total n and the sum of the n per gender was not explained by the authors.

d Quality of Life.

e World Health Organization Quality of Life – short version questionnaire.

f European Quality of Life Five Dimension Three Level Scale – questionnaire.

### 3.2 Study characteristics

The 12 articles were published between 1986 and 2021 and reported on nine distinct studies. All articles applied a cross-sectional design, with nine articles including qualitative measures, two articles administrating quantitative measures, and one article using mixed-method analysis. The quantitative and mixed-method studies included parents of severely ill children or grandparents of healthy children as comparison groups, while the qualitative studies did not include comparison groups (Table 1). The sample sizes varied from seven to 89 grandparents of severely ill children and from 50 to 134 comparison participants. Most studies were done in Canada (four articles reporting on three studies), followed by Brazil (two articles reporting on two studies), Australia and Denmark (each two articles reporting on one study), and USA and Sweden (one article each). The included articles focused on grandparents of children affected by childhood cancer (seven articles reporting on five studies), prematurely born babies (two articles reporting on two studies), children with Hypoplastic Left Heart Syndrome (HLHS; one article) and mixed diagnoses (two articles reporting on one study).

### 3.3 Synthesis of results

#### 3.3.1 Psychological outcomes

##### 3.3.1.1 Wide spectrum of emotions

Eleven articles reported on the wide range of feelings experienced by grandparents during their grandchild's disease. Grandparents' emotional journey was described as “living on a roller-coaster” and included a broad spectrum of emotions, going from worry and fear to anger and rage, or from hope to despair [17].

###### 3.3.1.1.1 Initial shock

When learning about the diagnosis, grandparents described being in shock [11, 14, 17, 18, 20, 23, 24] and feeling completely unprepared for the situation [18, 23]. When recalling the moment of getting to know about the diagnosis, they used expressions like “the earth had crumbled down” [14], “he was going to die, it was the only thought that came to our minds” [11], “it was an emotional experience” [23] or “universe shaker” [24].

###### 3.3.1.1.2 Emotional journey

After the initial shock, grandparents described feeling overwhelmed and helpless [11, 14, 18, 20]. These feelings emerged from the lack of control they had over the given situation and the illness of the child [12, 14, 18, 19, 20, 24]. Grandparents described difficulties in witnessing the child's suffering without being able to change the circumstances [11, 14, 18, 19, 20], felt angry towards the unjust position their grandchild was facing [14, 17] and questioned the fairness of the situation: “why not me?”, “little boys don't get cancer”, “I just don't think it's fair” [24] or “I find there is no justice on earth” [14]. In case of premature birth, grandparents showed ambivalent feelings, from joy and happiness of becoming grandparents to uncertainty about the future of the baby [19, 20, 23].

Moreover, grandparents found themselves in a state of perpetual waiting for news about their grandchild's health [19, 20] which led to experiencing frustration [11, 18, 19]. They oftentimes felt left outside of the information loop by the medical personnel and had to rely on second hand information provided by the parents [12, 18, 19, 20, 23, 24]. This situation made them feel unprepared for the child's disease and increased their suffering and feeling of isolation [11]. Furthermore, two studies compared quantitatively the levels of anxiety between grandparents of severely ill children and grandparents of healthy children and found that grandparents of unwell children scored significantly higher than comparisons on measurements of anxiety and anger [16, 17]. Additionally, when compared to parents of severely ill children, grandparents reported equal levels of anxiety and very similar degrees of fear and helplessness [18]. Generally, grandmothers experienced emotions stronger than grandfathers [18] and seemed more affected than their male partners [14], while grandfathers showed less burden to others than they actually experienced [20].

###### 3.3.1.1.3 Fears and worries

Despite the wide range of experienced emotions, the emotion most frequently reported in the included articles was fear. Grandparents identified various elements they were frightened of while their grandchild was ill. Mostly they worried about the chances of survival [14, 18, 19, 20, 23, 24, 25], the physical and/or the intellectual development [18, 19, 20] and the effects the treatment or the disease might have on the future health of their grandchild [11, 14, 18, 19, 20, 23]. These fears were expressed even after treatment had been completed or the child had been discharged from the hospital [19, 20]. In case of a diagnosis of cancer, grandparents were additionally concerned about the possibility of recurrence and became suspicious when the child showed any symptoms, such as cough or fever [11, 14]. At the same time, grandparents worried about their own children, the parents of the severely ill child [11, 12, 14, 15, 18, 19, 20, 23]. They wondered about parents' psychological and physical wellbeing [11, 14, 18, 19, 23], their ability to cope with the highly stressful situation [18, 20] and worried about the financial burden for the family due to the disease [18]. This phenomenon was identified in the literature as the “double concern” [19, 20]. In addition, grandparents’ fears were increased when the severely ill child had siblings. In this case, grandparents not only worried about the impact of the disease on the parents and the ill child, but were also worried about how the stressful situation would affect the adjustment and development of the healthy grandchildren [11, 14, 15, 18]. The combination of these three aspects was described as the “triple concern” [15]. However, apart from being worried about the other family members, some grandparents also expressed concerns for themselves and were worried about their own ability to cope with the given situation [11, 14, 18].

##### 3.3.1.2 Psychological well-being

Two publications investigated the psychological well-being of grandparents by administrating quantitative measures and comparing their scores to grandparents of healthy children. The results showed that grandparents of severely ill children scored significantly higher on distress, depression and need for help [17] and reached significantly lower levels of overall quality of life (QoL) and psychological health than the comparison group [16]. Grandparents of severely ill children also reported a higher consumption of medication against stress or anxiety when compared to grandparents of healthy children [16]. One study identified the gender of grandparents and the number of grandchildren as predictors for distress showing that grandmothers, but also grandparents with fewer grandchildren experienced higher levels of distress [17]. In a further publication, predictors for quality of life were investigated and the authors identified living in the city, being unemployed or retired, having a female grandchild, being a maternal grandmother and living further from the grandchild as predictors for lower QoL [16]. Generally, grandparents of severely ill children reveal higher deterioration of their psychological health [16], but at the same time they minimized the relevance of their well-being during the child's disease and perceived the well-being of parents and other family members as more important than their own [11, 23, 24].

##### 3.3.1.3 Family related emotional dynamic

During the treatment of the child, grandparents became more involved in the family system, developed stronger connections with their own children [11, 12, 16, 17, 24] and offered emotional care to the parents [11, 19, 25]. They felt proud about how well the siblings of the severely ill child adjusted to the difficult situation [15] and how well the parents were managing and coping with the stress [11, 12, 20, 24]. Grandparents reported that the disease of the child helped them feel emotionally closer to their own children and brought the family together [11, 12, 16, 19, 23, 24]. They also reported feeling a deep urge to be involved and help the family [14, 19, 20, 25]. Therefore, a big challenge for grandparents was to temper their urge to support and allowing for parents' desire of autonomy [11, 17, 19, 23, 24, 25]. They were alert, in case of being needed, but careful not to overstep and be intrusive. Occasionally, grandparents were criticized or blamed by the parents and felt that their actions were inappropriate [17, 24]. Besides the friction with parents, the stressful situation surrounding their grandchild's disease lead sometimes to challenging interactions and tense relationships among the grandparents, resulting in marital problems [12, 14, 16].

#### 3.3.2 Psychological support

##### 3.3.2.1 Informal support

Grandparents described that feeling supported by others was essential in being able to carry on, therefore they sought and received help from various parties. Some grandparents identified their spouses as the most important source of strength and support for coping with the given situation [12, 14, 18]. Other grandparents shared their pain and suffering with their own children as they supported each other throughout the crisis, leading to a mutual helping dynamic within the family [11, 19, 20, 25]. Yet, while certain grandparents shared the burden with their own children, others were afraid of increasing the pain of the parents, so they deliberately requested comfort and support from extended family members, friends or colleagues [11, 12, 14, 23].

Grandparents identified the parents’ suffering as the greater possible burden and did not see their own needs and pain as being important: “What suffering can be greater than that of the parents?” [11]. In order to not overload their own children and create more aggravation, some grandparents decided to suffer in silence. Accordingly, they suppressed their feelings in front of the parents, avoided crying or expressing their worries when being around the parents and did not talk about certain topics with their children [11, 12, 14, 24]. Furthermore, a small number of grandparents mentioned they had no one to rely on or talk to and felt uncomfortable to discuss their concerns and struggles with their partners, children or friends [12, 24].

##### 3.3.2.2 Formal support

Despite the overwhelming variety of feelings grandparents experienced and their need for support, they did not access or rarely asked for professional psychological help [17, 23]. Some grandparents highlighted that there were no services available to them: “It would have been nice, if we could have gone somewhere and dumped… a psychologist or something” [12]; “If they want to help grandparents, maybe they should have something for them… I don't know what… something we could have gone to” [12]. Grandparents pointed out the lack of infrastructure and stated that visiting a support group could have had a cathartic role throughout their grandchild's disease: “A place to go and break down… and then go back to their kids to be a rock” [24]. In terms of obstacles to accessing formal support, one study identified lack of knowledge and geographical isolation as barriers to access professional psychological support [17]. In general grandparents did not use formal support and chose to ask non-professionals for emotional help or coped on their own with the situation.

##### 3.3.2.3 Access to information

Grandparents described struggling with their lack of information and reported needing to know more in order to better handle the stressful circumstances. On the one hand they were not familiar with the disease of the child and on the other hand they were not always updated about the health development of their grandchild. Getting educated about the child's condition helped grandparents to understand the nature of the illness, adapt their expectations to reality and reduced their worries [14, 23]. Furthermore, being contacted by the parents and receiving details about the child's health status, helped them to better cope with the uncertainty of the situation [20, 23]. Grandparents expressed their support in creating an information booklet specially tailored to their situation and believed in the necessity of being better informed in order to handle properly the challenging situation: “The biggest thing back then is that we knew so little. If there had been more information and more options and there was nowhere to talk”; “Maybe even a pamphlet at the [hospital] that says: Here! Other people are going through this” [12]. Being afraid of creating additional pain with their questions, grandparents were cautious about asking parents for information and used the internet to gather knowledge and educate themselves [14, 23].

##### 3.3.2.4 Faith

Grandparents emphasized the importance of having hope that the child will survive in order to move forward and manage the difficult circumstances. They adopted a hopeful and positive attitude for their own sake, but also in order to empower the overwhelmed parents [14, 19, 20]. Having faith in god, going to the church and/or praying gave grandparents the needed comfort and confidence for the future [12, 14, 17, 25]. Besides the spiritual faith, believing in the competences of the medical team and relying on the progress of medicine reduced grandparents’ worries and gave them assurance for the recovery of the child [14, 20, 23].

## 4 Discussion

The objectives of the current systematic review were to identify the psychological outcomes experienced by grandparents who have a grandchild suffering from a severe physical illness and to describe what psychological support was needed and used by grandparents in relation to their psychological outcomes. We found that grandparents experienced a wide spectrum of emotions, especially fear, and worried for each member of the family. Grandparents' psychological well-being was impaired as a result of their grandchild's health problems, but they did not think of their psychological health as a priority and considered the well-being of the family more important than their own. Despite their psychological problems, grandparents did not make use of any formal psychological support due to the inexistence of formal support services addressing grandparents or due to their lack of knowledge about the available services. In consequence, grandparents limited themselves by asking and receiving support from non-professionals, i.e. friends, family members, neighbors, work colleagues or the church community.

### 4.1 Psychological outcomes

#### 4.1.1 Emotional experiences

Grandparents went through a roller-coaster of emotions and encountered a variety of alternating feelings such as shock, disbelief, faith, anxiety, anger, helplessness, hope, sadness, frustration, overwhelm. The emotions experienced by grandparents were very much alike to the ones described by the parents [3, 18]. The similarity between parents and grandparents shows the severity and the impact the illness of a child has on their grandparents.

Regardless of the broad spectrum of emotions named by grandparents, fear was the emotion most frequently reported throughout the analyzed articles. We found that grandparents of seriously ill children were afraid for their grandchild's life, the effects of the treatment on the future development of the child, and their psychological well-being. Furthermore, grandparents have expressed concerns about the mental and physical health of their own children – the parents of the ill grandchild – and at the same time they were afraid about how the stressful situation would affect the development of the siblings of the ill child. The multiple fears experienced by grandparents when worrying about the ill grandchild, the parents, but also the siblings of the ill child was introduced in the literature as the concept of the “third concern” [15]. In addition, we identified a further concern described by grandparents, i.e. their worries about their own strength and ability to cope with the overwhelming situation [11, 14, 18]. This last aspect shows the multidimensional struggle grandparents of ill children experience and it adds another layer to the impact of having a severely ill grandchild. We understand the delicate in-between position of the grandparents as a position that generates *multiple concerns*, with grandparents suffering for the ill child, the parents, the siblings, the family in general and for themselves.

#### 4.1.2 Gender differences in grandparents

Grandmothers reported being more affected by the experienced emotional roller-coaster than grandfathers [14] and it was shown that they presented more distress and lower QoL than their male partners [16, 17]. This aspect mirrors the position of parents of severely ill children, where mothers tend to show more psychological burden, higher stress and anxiety levels, but also more post-traumatic stress symptoms and burnout symptoms than fathers [2, 4, 6, 26, 27]. Furthermore, grandfathers revealed that their emotional experiences were intense, but they deliberately chose to show less feelings and not express their struggles [20]. This highlights the need of gender specific services and shows the lack of interventions specially tailored to grandparents' necessities. Socially men are expected to a lesser degree to express their feelings, and thus tend to show less emotions than women [28]. It is important to acknowledge and consider the social expectations in expressing and living emotions for each gender, and allow for both women and men to express their pain. Comprehending men's emotions and supporting them in understanding their own feelings could improve their well-being and health outcomes [29]. Acknowledging these aspects and providing support accordingly will hopefully prepare both grandmothers and grandfathers to better cope with the psychological difficult situation and in return will help them to care better for the other family members.

### 4.2 Psychological support

#### 4.2.1 Informal vs. formal support

Our analysis has shown that grandparents have rarely used professional help and rather coped on their own with the stress and emotions or requested support from external family members, friends and acquaintances. Despite being highly affected by their grandchild's illness, grandparents not only did not access professional help, but they considered their feelings and needs to be less important than those of the other family members and did not make a priority out of their well-being. In addition, living in a geographically isolated area or not knowing that formal care is available were two aspects that have hindered grandparents from seeking professional support [17]. These findings show that there is a need to guide and inform grandparents on how and where to get the proper support and emphasize the missing infrastructure for services addressing their situation. It is important to acknowledge the suffering of all family members, including grandparents, and help them understand that they have the right to suffer and give them the space and opportunity to express and work with their emotions and psychological burden.

#### 4.2.2 Importance of information

Apart from not knowing where to find professional support, grandparents also struggled with not knowing enough about their grandchild's illness and not always being updated about the health development of the child. This dual informational shortcoming produced extra uncertainty and frustration for grandparents and enhanced their fears and worries. Parents were found to value easily accessible and repeated information, e.g. in a simple app format [30]. Presumably, clear information formats could also reduce uncertainties and anxiety among grandparents, who expressed the necessity of being properly informed in order to handle better the challenging situation and to know how to assist the family [12, 23, 31]. This would help to provide grandparents with the necessary information about the child's condition and health state, and support building realistic expectations about the grandchild's future prognosis and reducing their worries by understanding the natural course of the illness.

### 4.3 Clinical and research implications

Understanding the emotional struggle of grandparents of severely ill children and their support needs has so far been an underexplored area of research. The current review shows the limited number of publications focusing on this group and underlines the need for research on these so far forgotten members of the family. Addressing the research gap will lead to a direct impact on the life of the affected families and all their members. Moreover, our findings underline the absence of professional services available to all members of families struggling with an ill child and show the necessity of thinking and developing interventions that are specially tailored for grandparents’ emotional burden and suffering. Grandparents themselves stated that they were consoled by knowing that other people were going through a similar experience [20, 25], therefore developing support groups where grandparents can meet and exchange experiences with other grandparents facing comparable situation, might lower their psychological burden and help them deal with the variety of the overwhelming emotions. Having a female grandchild, being a maternal grandmother, being unemployed or retired, having fewer grandchildren, living in a major city or further from the grandchild were identified as predictors for worse psychological health in grandparents. Addressing these elements in a family centered approach and informing families about the risk factors, but also providing them with professional support services, will lead to a reduction in stress, anxiety and other poor psychological outcomes across the entire family unit.

### 4.4 Limitations and strengths

In our review, we only included self-reported data, which might have led to biased responses due to social desirability with grandparents describing the experiences of their grandchild illness in a more selective way. Research was available mostly from English speaking countries, therefore our results cannot be generalized to countries and cultures from Europe, Asia or Latin America. A major strength of our study is the methodological approach used in the review. We searched the most relevant available databases in the field, we used an extensive list of search terms developed in an interdisciplinary team of experts, and all analyses and evaluations were conducted by two independent researchers. In addition, we applied no restrictions in terms of study designs, allowing our study to assess and answer the research equations by integrating qualitative and quantitative measures.

## 5 Conclusion

Having a child with a severe illness leads to psychological challenges in all family members, including grandparents. Grandparents are an active and highly involved part of the family system and the current review shows that they are deeply impacted by their grandchild's illness. Despite being highly affected, grandparents primarily relied on informal support from non-professionals and did not access or did not know where to access formal support. They reported similar emotional experiences to those of the parents and struggled with their lack of information about the child's illness. We conclude that providing grandparents with understandable information about their grandchild's disease and familiarizing them with the available support services, might help reducing their psychological burden and allow them to better attend the needs of the other family members.

## Declarations

### Author contribution statement

All authors listed have significantly contributed to the development and the writing of this article.

### Funding statement

This work was supported by 10.13039/501100001711Schweizerischer Nationalfonds zur Förderung der Wissenschaftlichen Forschung [10001C_182129/1].

### Data availability statement

Data included in article/supp. material/referenced in article.

### Declaration of interests statement

The authors declare no conflict of interest.

### Additional information

No additional information is available for this paper.

## Figures and Tables

**Appendix Table 1:** Blocks and search terms used in searching the four electronic databases

	PubMed	PsycINFO	Scopus	CINAHL
**Block 1:** **Child**	**child** OR child∗ OR kid∗ OR baby∗ OR **infant** OR infant∗ OR newborn∗ OR neonat∗ OR pediatric∗ OR paediatric∗ OR girl∗ OR boy∗ OR toddler∗ OR “pre-schooler” OR preschooler∗ OR “pre schooler” OR “pre schoolers” OR **adolescent** OR adolescen∗ OR young∗ OR youth∗ OR teen∗	child∗ OR kid∗ OR baby∗ OR infant∗ OR newborn∗ OR neonat∗ OR pediatric∗ OR paediatric∗ OR girl∗ OR boy∗ OR toddler∗ OR “pre-schooler” OR preschooler∗ OR “pre schooler” OR “pre schoolers” OR **adolescent health** OR adolescen∗ OR young∗ OR youth∗ OR teen∗	child OR child∗ OR kid∗ OR baby∗ OR infant OR infant∗ OR newborn∗ OR neonat∗ OR pediatric∗ OR paediatric∗ OR girl∗ OR boy∗ OR toddler∗ OR {pre-schooler} OR preschooler∗ OR {pre schooler} OR {pre schoolers} OR adolescent OR adolescen∗ OR young∗ OR youth∗ OR teen∗	**child** OR child∗ OR kid∗ OR baby∗ OR **infant** OR infant∗ OR newborn∗ OR neonat∗ OR pediatric∗ OR paediatric∗ OR girl∗ OR boy∗ OR toddler∗ OR “pre-schooler” OR preschooler∗ OR “pre schooler” OR “pre schoolers” OR **adolescent health** OR adolescen∗ OR young∗ OR youth∗ OR teen∗
**Block 2:** **Grandparent**	**grandparents** OR grandparent∗ OR “grand parent” OR “grand parents” OR grandmother∗ OR “grand mother” OR “grand mothers” OR grandfather∗ OR “grand father” OR “grand fathers” OR grandchild∗ OR “grand child” OR “grand children” OR granddaughter∗ OR “grand daughter” OR “grand daughters” OR grandson∗ OR “grand son” OR “grand sons”	**grandparents** OR grandparent∗ OR “grand parent” OR “grand parents” OR grandmother∗ OR “grand mother” OR “grand mothers” OR grandfather∗ OR “grand father” OR “grand fathers” OR grandchild∗ OR “grand child” OR “grand children” OR granddaughter∗ OR “grand daughter” OR “grand daughters” OR grandson∗ OR “grand son” OR “grand sons”	grandparents OR grandparent∗ OR {grand parent} OR {grand parents} OR grandmother∗ OR {grand mother} OR {grand mothers} OR grandfather∗ OR {grand father} OR {grand fathers} OR grandchild∗ OR {grand child} OR {grand children} OR granddaughter∗ OR {grand daughter} OR {grand daughters} OR grandson∗ OR {grand son} OR {grand sons}	**grandparents** OR grandparent∗ OR “grand parent” OR “grand parents” OR grandmother∗ OR “grand mother” OR “grand mothers” OR grandfather∗ OR “grand father” OR “grand fathers” OR grandchild∗ OR “grand child” OR “grand children” OR granddaughter∗ OR “grand daughter” OR “grand daughters” OR grandson∗ OR “grand son” OR “grand sons”
**Block 3:** **Psychological outcomes**	**stress disorders, traumatic** OR stress OR stress∗ OR “stress disorders” OR PTSD OR PTSS OR “post traumatic” OR “posttraumatic” **OR posttraumatic growth, psychological** OR posttraumatic growth OR psychological distress OR distress∗ OR **quality of life** OR “quality of life” OR QOL OR “life quality” OR “health related quality of life” OR HRQOL OR well-being OR wellbeing OR wellbeing∗ OR psycholog∗ OR **depression** OR depress∗ OR concern∗ OR **anxiety** OR anxiet∗ OR worry∗ OR worri∗ OR **fear** OR fear∗ OR **emotions** OR “emotional experience” OR “emotional experiences” OR **sadness** OR **happiness** OR joy∗ OR positiv∗ OR negativ∗ OR **hope** OR hope∗ OR **health** OR **mental health** OR “psychological health” OR “psychological outcomes”	**stress** OR stress∗ OR “stress disorders” OR **acute stress disorder** OR **posttraumatic stress disorder** OR PTSD OR PTSS OR “post traumatic” OR “posttraumatic” OR **posttraumatic growth** OR **distress** OR distress∗ OR “psychological distress” OR **quality of life** OR “quality of life” OR QOL OR “life quality” OR **health related quality of life** OR “health related quality of life” OR HRQOL OR **well being** OR well-being OR wellbeing OR wellbeing∗ OR psycholog∗OR **depression (emotion)** OR depression OR depress∗ OR concern∗ OR **anxiety** OR **anxiety disorders** OR anxiet∗ OR worry∗ OR worri∗ OR **fear** OR fear∗ OR **sadness** OR **happiness** OR joy∗ **hope** OR **hopelessness** OR hope∗ OR **emotions** OR **emotional states** OR “emotional experience” OR “emotional experiences” OR positiv∗ OR negativ∗ OR **mental health** OR “psychological health” OR “psychological outcomes”	stress disorder OR stress OR stress∗ OR {stress disorder} OR PTSD OR PTSS OR {post traumatic} OR {posttraumatic} OR posttraumatic growth OR psychological distress OR distress∗ OR quality of life OR {quality of life} OR QOL OR {life quality} OR {health related quality of life} OR HRQOL OR well-being OR wellbeing OR wellbeing∗ OR psycholog∗ OR depression OR depress∗ OR concern∗ OR anxiety OR anxiet∗ OR worry∗ OR worri∗ OR fear OR fear∗ OR emotions OR {emotional experience} OR {emotional experiences} OR sadness OR happiness OR joy∗ OR positiv∗ OR negativ∗ OR hope OR hope∗ OR health OR mental health OR {psychological health} OR {psychological outcomes}	**psychological stress** OR stress OR stress∗ OR “stress disorders” OR **POST-traumatic stress** OR PTSD OR PTSS OR “post traumatic” OR “posttraumatic” OR **posttraumatic growth** OR **distress** OR distress∗ OR **quality of life** OR “quality of life” OR QOL OR “life quality” OR “health related quality of life” OR HRQOL OR **psychological well-being** OR wellbeing OR wellbeing∗ OR psycholog∗ OR **mental depression** OR depression OR depress∗ OR concern∗ OR **anxiety** OR anxiet∗ OR worry∗ OR worri∗ OR **fear** OR fear∗ OR **sadness** OR happiness OR joy∗ OR **hope** OR hope∗ OR **emotions** OR “emotional experience” OR “emotional experiences” OR positiv∗ OR negativ∗ OR **health** OR **mental health** OR “psychological health” OR “psychological outcomes”
**Block 4:** **Severe** illness	**neoplasms** OR neoplasm∗ OR cancer∗ OR malign∗ OR tumor∗ OR tumour∗ OR oncolog∗ OR carcinoma∗ OR leukemia∗ OR leukaemia∗ OR lymphoma∗ OR medulloblastoma∗ OR sarcom∗ OR **radiotherapy** OR radiotherap∗ OR **chemoradiotherapy** OR **chemotherapy, adjuvant** OR **induction chemotherapy** OR **consolidation chemotherapy** OR **maintenance chemotherapy** OR chemotherap∗ OR **surgical oncology** OR “surgical oncology” OR “curative surgery” OR **chronic disease** OR **genetic disease, inborn** OR “genetic disease” OR **disease** OR disease∗ OR **critical illness** OR illness∗ OR life threat∗ OR disability OR disabilit∗ OR disable∗ OR **disabled children** OR “sick children” OR **congenital** OR congenital∗ OR connatal∗ OR **cardiovascular diseases** OR “congenital heart disease” OR cardiolog∗ OR **nervous system diseases** OR “neurological disease” OR neurologic∗ OR somatic∗ OR **physiopathology** OR **cystic fibrosis** OR mucoviscidosis∗ OR **meningitis** OR meningitis∗ OR **hospitalization** OR hospitalization∗ OR **accidents** OR **wounds and injuries** OR **brain injuries** OR **brain diseases** OR cerebral∗ OR pulmonary∗ OR **lung diseases** OR **syndrome** OR syndrom∗ OR **premature birth** OR **spinal cord diseases** OR “spinal cord injury” OR “spinal cord disease” OR myelopath∗ OR SCI OR **spinal dysraphism** OR “spina bifida” OR **epilepsy** OR epileps∗ OR **inflammatory bowel diseases** OR **Crohn disease** OR “Morbus Crohn” OR c**olitis** OR "Colitis Ulcerosa"	**neoplasms** OR neoplasm∗ OR cancer∗ OR malign∗ OR tumor∗ OR tumour∗ OR oncolog∗ OR carcinoma∗ OR leukemia∗ OR leukaemia∗ OR lymphoma∗ OR medulloblastoma∗ OR sarcom∗ OR **radiology** OR radiotherap∗ OR **chemotherapy** OR chemotherap∗ OR “surgical oncology” OR “curative surgery” OR **chronic illness** OR **genetic disorders** OR “genetic disease” OR disease OR disease∗ OR “critical illness” OR illness∗ OR **chronically ill children** OR life threat∗ OR disability OR disabilit∗ OR disable∗ OR disabled children OR “sick children” OR **congenital disorders** OR congenital∗ OR connatal∗ OR **cardiovascular disorders** OR “congenital heart disease” OR cardiolog∗ OR **nervous system disorders** OR “neurological disease” OR neurologic∗ OR somatic∗ OR physiopathology OR **cystic fibrosis** OR mucoviscidosis∗ OR **meningitis** OR meningitis∗ OR **hospitalization** OR hospitalization∗ OR accidents OR **injuries** OR **brain injuries** OR **brain disorders** OR cerebral∗ OR pulmonary∗ OR **lung disorders** OR syndrome OR syndrom∗ OR **premature birth** OR **spinal cord injuries** OR “spinal cord injury” OR “spinal cord disease” OR myelopath∗ OR SCI OR spinal dysraphism OR **spina bifida** OR “spina bifida” OR **epilepsy** OR epileps∗ OR inflammatory bowel diseases OR Crohn disease OR “Morbus Crohn” OR **colitis** OR "Colitis Ulcerosa"	neoplasms OR neoplasm∗ OR cancer∗ OR malign∗ OR tumor∗ OR tumour∗ OR oncolog∗ OR carcinoma∗ OR leukemia∗ OR leukaemia∗ OR lymphoma∗ OR medulloblastoma∗ OR sarcom∗ OR cancer radiotherapy OR radiotherap∗ OR chemoradiotherapy OR cancer chemotherapy OR chemotherap∗ OR cancer surgery OR {surgical oncology} OR {curative surgery} OR chronic disease OR genetic disease OR {genetic disease} OR disease OR disease∗ OR critical illness OR illness∗ OR life threat∗ OR disability OR disabilit∗ OR disable∗ OR disabled children OR {sick children} OR congenital OR congenital∗ OR connatal∗ OR cardiovascular diseases OR {congenital heart disease} OR cardiolog∗ OR nervous system diseases OR {neurological disease} OR neurologic∗ OR somatic∗ OR physiopathology OR cystic fibrosis OR mucoviscidosis∗ OR meningitis OR meningitis∗ OR hospitalization OR hospitalization∗ OR accidents OR wounds and injuries OR brain injuries OR brain diseases OR cerebral∗ OR pulmonary∗ OR lung diseases OR syndrome OR syndrom∗ OR premature birth OR spinal cord diseases OR {spinal cord injury} OR {spinal cord disease} OR myelopath∗ OR SCI OR spinal dysraphism OR {spina bifida} OR epilepsy OR epileps∗ OR inflammatory bowel diseases OR Crohn disease OR {Morbus Crohn} OR colitis OR {Colitis Ulcerosa}	**neoplasms** OR neoplasm∗ OR cancer∗ OR malign∗ OR tumor∗ OR tumour∗ OR oncolog∗ OR carcinoma∗ OR leukemia∗ OR leukaemia∗ OR lymphoma∗ OR medulloblastoma∗ OR sarcom∗ OR **radiotherapy** OR radiotherap∗ OR **chemoradiotherapy** OR **chemotherapy, cancer** OR chemotherap∗ OR “surgical oncology” OR “curative surgery” OR **chronic diseases in infants** OR **chronic diseases in children** OR **chronic diseases in adolescence** OR **genetic disorders** OR “genetic disease” OR disease OR disease∗ OR **critically ill** OR illness∗ OR life threat∗ OR **disabilities** OR disabilit∗ OR disable∗ OR **children with disabilities** OR **sick children** OR **human abnormalities** OR congenital OR congenital∗ OR connatal∗ OR **cardiovascular diseases** OR **congenital heart disease** OR “congenital heart disease” OR cardiolog∗ OR **nervous system abnormalities** OR “nervous system disorder” OR “nervous system disease” OR neurological disorders OR neurologic∗ OR somatic∗ OR physiopathology OR **cystic fibrosis** OR mucoviscidosis∗ OR **meningitis** OR meningitis∗ OR **hospital care** OR hospitalization∗ OR **accidents** OR **chronic wounds & injuries** OR **brain injuries** OR **brain diseases** OR cerebral∗ OR pulmonary∗ OR **lung diseases** OR syndrome OR syndrom∗ OR **premature labor** OR **spinal cord diseases** OR “spinal cord injury” OR “spinal cord disease” OR myelopath∗ OR SCI OR spinal dysraphism OR **spina bifida** OR **epilepsy** OR epileps∗ OR **inflammatory bowel diseases** OR **Crohn's disease** OR “Morbus Crohn” OR **colitis** OR "Colitis Ulcerosa"
**Search strategy**	Block1 AND Block2 AND Block3 AND Block4			

Note: Words written in bold represent the identified MeSH terms.
